# The Role of Cysteine Proteinases and their Inhibitors in the Host-Pathogen Cross Talk

**DOI:** 10.2174/138920312804871102

**Published:** 2012-12

**Authors:** Nataša Kopitar-Jerala

**Affiliations:** Department of Biochemistry, Molecular and Structural Biology, »Jožef Stefan« Institute, Jamova 39, 1000 Ljubljana, Slovenia

**Keywords:** Bacteria, immune response, protease/cysteine proteinase, proteinase inhibitor, virus.

## Abstract

Proteinases and their inhibitors play essential functional roles in basic biological processes in both hosts and pathogens. Endo/lysosomal cathepsins participate in immune response in pathogen recognition and elimination. They are essential for both antigen processing and presentation (host adaptive immune response) and activation of endosomal Toll like receptors (innate immune response). Pathogens can produce proteases and also natural inhibitors to subvert the host immune response. Several pathogens are sensed through the intracellular pathogen recognition receptors, but only some of them use the host proteolytic system to escape into the cytosol. In this review, I provide an update on the most recent developments regarding the role of proteinases and their inhibitors in the initiation and regulation of immune responses.

## 1 INTRODUCTION 

Multicellular organisms have continuous interactions with both pathogenic and non-pathogenic microbes and have developed a set of antimicrobial recognition and defense systems, which enable them to survive. The innate immune system detects molecular structures, denoted as pathogen associated molecular patterns (PAMPs) that are distinct from host molecular pattern and are frequently found in bacteria, fungi, virus, and some protozoans. These PAMPs are detected using an array of pattern-recognition receptors (PRRs) [1-3], which are expressed at the first line of defense against infection by cells like macrophages, monocytes, dendritic cells, neutrophils and epithelial cells, as well as cells of the adaptive immune system [3]. PRRs include the membrane-bound Toll-like receptors (TLRs), the cytosolic NOD like receptors (NLRs) and the RNA-sensing RIG-like helicases (RLHs) [3]. The outcome of PAMP recognition by PRRs leads to signal transduction from these receptors which converges on a common set of signaling modules, often including the activation of the NF-κB and AP-1 transcription factors that drive proinflammatory cytokine/chemokine production [4,5]. 

Ligand recognition by TLRs is mediated by the extracellular or ectodomains that contain 19 to 25 leucine-rich repeat (LRR) motifs, a transmembrane domain and a cytoplasmic TIR domain [6-8]. Structural and biochemical studies revealed that all TLRs form either hetero or homodimers (e.g., TLR1/TLR2, TLR2/TLR6, TLR3/TLR3, and TLR4/TLR4) [7,9]. In mammals, 13 TLR family members have been described (13 TLR in mice and 11 TLR in humans) [10]. While TLR1, 2, 4, 5 and 6 are primarily expressed on the cell surface and recognize PAMPs derived from bacteria, fungi and protozoa, TLR4 recognizes lipopolysaccharide (LPS), a major cell wall component of gram-negative bacteria [11-14]. An essential component of gram-positive bacteria, peptidoglycan is sensed by TLR2 [15], which also detects lipoarabinomannan (LAM) of Mycobacteria [16]. TLR2 could form also heterodimers (in conjugation with TLR1 or TLR6) and as a heterodimer recognizes diacyl or triacyl lipopeptides on bacteria, mycobacteria and mycoplasma. TLR5 senses the flagellin protein expressed by flagellated bacteria [1]. TLR6 participates in the recognition of macrophage-activating lipoprotein 2 kD (MALP-2) derived from mycoplasma [17]. The intracellular TLR3, TLR7, TLR8, and TLR9 are localized on the ER membrane and only upon stimulation with PAMPs, they are targeted into the endosomes [18]. The intracellular localization of TLR3, TLR7, TLR8, and TLR9 is regulated by the ER membrane protein UNC93B, which directly interacts with the intracellular TLRs [19]. TLR9 recognizes genomic DNA from DNA viruses such as HSV-1, HSV-2, or MCMV [1]. Viral single-stranded RNAs (ssRNAs) derived from HIV or influenza virus are recognized by TLR7 [3]. TLR3 recognizes dsRNA derived from Reoviruses and a synthetic double-stranded RNA (dsRNA) analog, polyinosinic-polycytidylic acid (poly I:C) [3]. Signaling through TLR1, TLR2, TLR4, TLR5 and TLR6 primarily induces the production of inflammatory cytokines, whereas TLR7 and TLR9 induce type I interferons (IFN) [5]. Recently, several excellent reviews have described the signaling of innate immune receptors, therefore those aspect will not be discussed in detail [4-6,20]. 

Proteases play significant roles in innate as well as adaptive immune response. They are classified by their gene sequence homology and according to their catalytic mechanism as cysteine, serine, threonine, aspartate and metalloproteases, or unknown proteases [21] (http://www.merops.sanger.ac.uk). The activity of different proteases is tightly controlled at the level of expression, zymogene activation, localization in different cellular compartments, or by protease inhibitors. 

Many pathogenic organisms synthesize proteases that resemble host proteases. Indeed, amino-acid sequences typical of pathogen proteases exist in key molecules involved in host immune response regulation including immunoglobulins (Ig), cytokines and chemokines. Here, I will discuss the functional role of proteinase and inhibitors from metazoan hosts and microbial pathogens, at the cross talk of host pathogen interactions and the contribution of these interactions to host protection and susceptibility to infections.

## 2 PAPAIN LIKE CYSTEINE CATHEPSINS

### 2.1 General Features of Cysteine Cathepsins 

Cysteine cathepsins were long known to be involved in protein degradation in lysosomes [22]. They are papain-like cysteine proteinases belonging to clan CA, family C1 [23]*.* Eleven human cathepsins are known (B, H, L, S, C, K, O, F, V, X and W). With the exception of cathepsins S, V, K and W, they are widely expressed in a number of different cells and tissues. Despite similarities in sequence and structure, cysteine cathepsins differ among each other in specificity. Most of the cathepsins are endopeptidases, although cathepsin B and X are also carboxydipeptidases, and cathepsin H and C are aminopeptidases [24,25]. Cysteine cathepsins exhibit a broad variety of functions [26-28]. The human genome encodes for two cathepsin L-like proteases, namely the human cathepsin L and cathepsin V (cathepsin L2), whereas in mouse only cathepsin L is present [29]. Cathepsin V expression is restricted to thymus, testis and corneal epithelium, while cathepsin L is ubiquitously expressed [30,31]. Cathepsins are synthesised as preproproteins, which are activated either by other proteinases or self-activated (in the case of endopeptidases). Cathepsins are optimally active in the acidic environment in endolysosomes. However, they are still active in the extracellular space and in the nucleus despite a neutral pH [32]. Seminal study by Goulet *et al.* showed that nuclear procathepsin L processed the transcriptional factor CUX1 into a form with enhanced DNA binding and that promotes cell cycle progression [32]. Cathepsin L was targeted into the nucleus through translation initiation at alternative start codons downstream of the normal signal sequence [32]. Recently, also cathepsin B and F were reported to be localized in the nucleus [33-35]. Our recent work demonstrated that the activity of cathepsin L in the nucleus is regulated by a nuclear cystatin, denoted as stefin B [36]. The regulation of nuclear cathepsin F activity by stefin B in hepatic stellate cells was involved in the transcriptional regulation of two activation markers and implies the role of stefin B in transcriptional regulation [34]. 

### 2.2 Endogenous Protein Inhibitors of Cysteine Cathepsins

The activity of cathepsins is regulated by interaction with their endogenous protein inhibitors: the cystatins [37-39], thyropins [40] and some of the serpins [41]. Thyropins are a superfamily of inhibitors homologous to the thyroglobulin type-1 domains [40]. The best characterized human representative so far is the MHC-class II associated invariant chain (Ii) fragment, which strongly inhibits cathepsin L and cruzipain [42-44]. Cystatins are reversible and tight-binding inhibitors of papain (C1) and legumain (C13) families of cysteine proteases and are characterized by a strong sequence and structure conservation [45]. The tertiary structures of cystatins are conserved and exhibit the so called “cystatin fold”, which is formed by a five stranded anti-parallel β-sheet wrapped around a five-turn α-helix [46,47]. The cystatin family I25 contains three subfamilies: I25A, B and C, as defined in the MEROPS database of protease and protease inhibitor information (http://merops.sanger.ac.uk/) [21]. Cystatins are found in plants, fungi and animals as well as in viruses. Type 1 cystatins, denoted as stefins, are predominantly present in the cytosol and the nuclei, while Type 2 cystatins are mainly extracellular, secreted proteins. These latter are synthesized with 20-26 residue long signal peptides, most of them found in physiologically relevant concentrations in body fluids. Type 3 cystatins are multidomain proteins of high molecular mass (60-120 kDa) and present three tandemly repeated type 2-like cystatin domains [48]. The mammalian cystatins belonging to this type are called kininogens [49], which were first known as kinin precursor proteins. The serpins are essentially serine proteinase inhibitors [50,51], only some of them inhibit both serine and cysteine proteases [41]. The mechanism by which cysteine proteases are inhibited involves the cleavage of the serpin, in some cases involving a stable covalent complex [52-54] and in other cases not [55].

## 3 CYSTEINE CATHEPSINS AND INHIBITORS IN THE CELLS AND TISSUES OF THE HOST

### 3.1 Macrophages 

Macrophages play a critical role in host defense against pathogens and are present in virtually all tissues [56]. They can change their physiology in response to micro-environmental stimuli. Classically activated macrophages or M1, primed with IFN-γ and stimulated with LPS, are involved in inflammatory responses to bacterial and viral infection [57]. Stimulation of macrophages with the cytokines interleukin 4 (IL-4) or IL-13 induces alternatively activated (called M2) macrophages [58-60]. The M2 macrophages include several types of activated macrophages, not only wound healing macrophages, but also regulatory macrophages and tumor-associated macrophages. Regulatory macrophages can secrete large amounts of interleukin-10 (IL-10) in response to Fc receptor-γ ligation [61,62]. 

M1 macrophages produce high amounts of proinflammatory cytokines, such as tumor necrosis factor alpha (TNF-α), upon recognition of invading pathogens by a set of PRRs including TLRs, RLRs and NLRs. M1 macrophages are known to produce nitric oxide (NO) by expressing inducible NO synthase (iNOS) and are critical for clearing bacterial, viral and fungal infections. Early studies reported that macrophages activated with IFN-γ and stimulated with IFN-γ chicken cystatin generated increased amounts of NO and the cytokines TNF-α and interleukin 10 (IL-10), in comparison with macrophages activated only with IFN-γ [63,64]. Experiments on macrophages prepared from cystatin C-deficient mice revealed that IFN-γ-primed cystatin C-deficient macrophages exhibit significantly higher IL-10 but lower TNF-α expression, compared to similarly primed wild type macrophages [65].

Upon the classical activation mechanism, macrophages up-regulate a variety of proteinases that can degrade endocytosed pathogens and play a critical role in antigen processing and presentation. The role of endosomal cathepsins has been described in several extensive reviews [66,67]. Only some of the endogenous inhibitors (cystatin F and Spia3g) are also up regulated, while cystatin C is down regulated [65,68]. Serpina3g (Spia3g) is highly induced in macrophages during bacillus Calmette-Guérin infection as well as in infection with *Salmonella typhimurium* and *Listeria monocytogenes *[68]. It was demonstrated that a ubiquitin homolog, IFN-stimulated gene of 15-kDa (ISG15) is conjugated to Spia3g in activated macrophages. It was reported that the stimulation of murine macrophages with IFN-γ resulted in increased cathepsin S activity and down-regulation of intracellular cathepsin L activity, despite the persistence of high levels of mature cathepsin L protein [69]. The reason for the lack of cathepsin L enzyme activity is still not clear. Beers *et al.* showed that inhibitors of cysteine proteinases cystatin C and p41 form of major histocompatibility complex invariant chain did not inhibit cathepsin L and the authors suggested that cystatin F might be the inhibitor that selectively regulated cathepsin L activity in macrophages [69]. Recently, Colbert *et al.* found equivalent loss of cathepsin L activity in IFN-γ stimulated wild type and cystatin F-deficient macrophages, indicating that cystatin F did not inhibit cathepsin L activity in activated macrophages [70]. We showed that cathepsin L is targeted to the nucleolus of classically activated (M1), but not un-stimulated and alternatively activated (M2) macrophages [71]. Therefore, we proposed that lack of activity of cathepsin L in classically activated macrophages could be, at least in part, due to different nucleolar localization of cathepsin L and co-localization with Spia3g [71]. Since only the pro-inflammatory stimuli (IFN-γ and LPS) and not the anti-inflammatory stimuli (IL-4) induce increased nucleolar localization of Spia3g, it is possible that Spia3g functions in the nucleolus are important for the host defense against pathogens. Spia3g is a mouse specific serpin and a human homologue has not been described so far, therefore it is not clear which protein compensates for Spia3g deficiency in human macrophages.

Inflammasomes are multiprotein complexes that activate caspase-1, an event which leads to maturation of the proinflammatory cytokines interleukin 1β (IL-1β) and IL-18 [72]. Cytosolic NLR (NLRP1, NLRP2, and NLRP3) are involved in assembly of inflammasome and the NLRP3 can be activated not only by bacteria, bacterial pore forming toxins or viruses [73], but also by a number of molecules like crystals silica, asbestos, alum and β-amyloid [74-78]. It was shown that cathepsin B (and possibly other cathepsins) leaking from the lysosomes had an important role in a direct NLRP3 activation by crystals and β-amyloid [74,75]. In addition, it was reported that live intracellular mycobacterium *M. kansasii* triggered the activation of the NLRP3 inflammasome and cathepsin B release from the endosomes and that the production of reactive oxygen species was essential in this process [79]. Therefore cysteine cathepins participate in the defense against pathogens in cytosol. The cytosolic cysteine proteinase inhibitors could regulate cathepsin activity in this process and prevent excessive inflammation.

### 3.2 Dendritic Cells (DC)

DCs are antigen presenting cells characterized by their efficient processing of internalized antigens and the presentation of peptide bound to major histocompatibility (MHC) complexes to the T cells [80,81]. Immature DCs reside in tissues and actively uptake antigens. Maturation of DCs can be achieved by TLRs [82-84]. The expression of a unique set of TLRs renders each type of DC susceptible to particular subsets of pathogens and the outcome of stimulation with TLR ligands can result in increased antigen uptake and presentation [85]. The population of DCs can be divided into 2 major sub populations: conventional DCs (cDCs) and plasmacytoid DCs (pDCs) [86]. While the macrophages contain high levels of endolysosomal proteases and rapidly degrade internalized antigens, cDCs express low levels of endolysosmal proteases and *in vivo* degrade internalized antigens slowly. However , the resulting limited lysosomal proteolysis in cDCs is favourable to the antigen presentation [87]. Subtle differences exist also among cDC; only cDCs isolated from peripheral blood or derived *in vitro* from CD34+ hematopoietic progenitor cells are protease poor, whereas cDCs differentiated *in vitro* from monocytes exhibit protease expression and activity similar to macrophages [88]. T lymphocytes recognize proteolytic fragments of antigens that are presented to them on MHC molecules. MHC class I molecules present primarily products of proteasomal proteolysis to CD8^+^ T cells, while MHC class II molecules display mainly degradation products of lysosomes for stimulation of CD4^+^ T cells [66]. MHC class II molecules are assembled in the endoplasmic reticulum with the assistance of chaperone invariant chain (Ii). A portion of Ii, termed CLIP, binds in the peptide groove of MHC class II molecules, thereby preventing premature loading of peptides [66]. Gene targeting studies showed a critical role for the lysosomal cysteine protease cathepsin S in the late stages of Ii degradation in B cells, DCs and macrophages [89-91] and cathepsin L (V in humans) in thymic cortical epithelium [92]. It has been proposed that cystatin C regulates the cleavage and removal of the MHC class II invariant chain (Ii) by regulating the activity of cathepsin S, and hence in the formation of MHC class II-peptide complexes [93]. Experiments on DC isolated from cystatin C-deficient mice showed that the lack of cystatin C did not change the formation of peptide-loaded MHC class II complexes in any of the DC types, nor the efficiency of antigen presentation [94]. Plasmacytoid DCs constitute a minor population of DCs; in the endolysosomes they express TLR7 and TLR9 and produce large amounts of IFN-α in upon TLR7 and TLR9 stimulation by viral nucleic acids [95]. Activated pDCs behave differently than conventional DCs in antigen presentation following activation via TLR9 ligands such as CpG DNA. In models of influenza infection, conventional DCs undergo maturation and present antigens in complex with MHC class II, with a parallel down-regulation of MHC II synthesis. Although pDCs also undergo maturation and present antigens, MHC class II molecules synthesis is not down-regulated upon stimulation with TLR ligands, indicating that the pDCs have the ability to continuously present viral antigens in activated state [83]. TLR recognition of viruses leads to IFN-α production, which positively feedbacks via interferon receptor to drive further type I IFN production by pDCs [82]. In the steady state, TLR3, TLR7 and TLR9 are mostly sequestered in the endoplasmic reticulum [18] and are transported into endosomes upon the activation with ligands [96,97]. An additional level of control is achieved by the proteolysis full length TLR7 and TLR9 by endosomal cysteine cathepsins and AEP.TLR9 can bind its ligand CpG DNA, but it cannot trigger activation signals without first being processed by endolysosomal proteases, which remove N-terminal region [98-101]. Cells derived from cathepsin B, L, K and S deficient mice show that no single protease is responsible for TLR7 or TLR9 processing, indicating that there is redundancy in this reaction [99,101]. The role of endogenous inhibitors in this process remains to be fully elucidated.

## 4 CYSTEINE CATHEPSINS AND THEIR INHIBITORS IN HOST PATHOGEN INTERACTIONS 

Many pathogens also synthesize cysteine proteases that act on target proteins in the host and thereby modulate host immune response. Pathogen-derived proteases range from nonspecific proteases that degrade multiple proteins involved in the immune response to enzymes that are very specific in their mode of action.

* Staphylococcus aureus* is the most frequently isolated pathogen in gram-positive sepsis. Among others, S. aureus secretes papain-like cysteine proteases: staphopain A (ScpA) and staphopain B (SspB). It was reported that enzymatically active staphopains degraded collagen and fibrinogen in the host, and the authors suggested that these activities could contribute in the clotting impairment and tissue destruction caused by staphylococcal infection [102]. Chemerin is a proinflammatory plasma protein that binds to the serpentine receptor CMKLR1 on macrophages and plasmacytoid dendritic cells and promotes chemotaxis [103]. It is secreted as a precursor protein and activated upon proteolytic cleavage of its C-terminus by serine proteases of the coagulation, fibrinolytic, and inflammatory cascades [104], as well as human cathepsins K and L [105]. SspB was reported to cleave and activate chemerin [106]. It was proposed that SspB may help to recruit pDC and macrophages at the site of infection and contribute to the ability of bacteria to elicit and maintain a chronic inflammation [106]. 

* In silico* studies revealed the presence of cystatin superfamily representatives in bacterial genomes and only some of them were pathogens of humans (*V. cholerae *and *V. vulnificu) *[107]. Since bacterial cystatins are homologues of eukaryotic ones, the authors of the analysis suggested that they might inhibit the cysteine proteases of their eukaryotic hosts [107]. Bacterial pathogen *Streptococcus pyogenes *secretes a highly specific protease, called Ides, which cleaves only immunoglobulin IgG [108,109]. Contrary to the expectations, IdeS activity is not inhibited by host protease inhibitors cystatin C, instead the protease activity was markedly stimulated. Kinetic studies revealed that the human cystatin C efficiently accelerated the enzymatic velocity of the pathogen cysteine protease IdeS and thus functions as a facultative cofactor for this bacterial protease [110]. Therefore more experimental data will be needed before we can make conclusions regarding the role of bacterial cystatins. Recently, it was reported that gram negative pathogen *Anaplasma phagocytophilum*, which causes human granulocytic anaplasmosis and is harboured within neutrophils, upregulates cathepsin L expression in the nucleus and leads to enhanced CUX1 cleavage and the repression of CUX1-regulated essential genes for effective neutrophil function [111]. 

The ways in which viruses entry the host cells are largely defined by the interactions between virus particles and their receptors at the host cell surface [112]. Enveloped viruses, such as orthomyxoviruses [113,114], paramyxoviruses [115] and retroviruses [116,117] encode proteins that mediate fusion of the viral envelope with target cell membranes, thus facilitating viral cell entry. The majority of viruses are internalized by endocytosis and delivered to the endosomes. In addition to host cell receptors, at least in some cases, endolysosomal cysteine cathepsins are required for their transport into the cytosol [118]. 

Filoviruses are enveloped, single-stranded, negative-sense RNA viruses. Infections by the Ebola and Marburg filoviruses cause a fatal haemorrhagic fever in humans and non human primates, for which no approved antivirals are available [119]. It has been shown that the endolysosomal processing by cathepsins B and L of the Ebola virus glycoprotein is essential for the delivery of viral material into the cytosol [118]. Ebola and Marburg entry into the host cell is mediated by the viral spike glycoprotein (GP), which attaches viral particles to the cell surface [120,121]. Ebola virus GP is synthesized as a single polypeptide chain that is cleaved in the Golgi into its receptor-binding (GP1) and fusion (GP2) subunits, which remain together through non-covalent interactions and through a disulphide bond. Three GP1-S–S-GP2 units come together to form the homotrimer that protrudes from the virion surface [120,121]. It was reported that the GP1 cleavage is a two-step event: first cathepsin L cleaved GP1 into 20 kDa fragment and after the cleavage with cathepsin B 19 kDa fragment is generated [122]. The translocation of pseudovirions bearing 20 kDa GP into cytoplasm was strongly inhibited by cathepsin B inhibitors, while the entry of pseudovirions bearing 19 kDa GP1 was not [123,124]. A recent study confirmed that GP cleavage by endosomal cathepsins was essential to reveal a putative binding domain for the endolysosomal cholesterol transporter Niemann–Pick C1 (NPC1) [125]. Lack of NPC1 on target cells prevented Ebola virus glycoprotein-dependent fusion [126]. Furthermore, it was reported that the virulence of only some of the Ebola virus species is strongly dependent on cathepsin B [127]. Ebola and Marburg viruses are highly pathogenic with mortality in humans up to 90% within days of exposure. While there are no FDA-approved vaccines or post-exposure treatment modalities available for preventing or managing Ebola virus or Marburg virus infections, several promising vaccines have been tested on nonhuman primates [128,129]. Considering the aggressive nature of Ebola infections, in particular the rapid and overwhelming viral burdens, early treatment with cathepsin inhibitors alone or in combination with neutralizing antibodies could help to bring down the number of death cases after infection. 

Not only Filoviruses, but also some of Coronaviruses, positive sense RNA viruses, were reported to entry the host cell by proteases dependent manner [130]. Human coronavirus 299E, a causative agent of the human common cold, enters cells via endosomes in which cathepsins are involved in the fusogenic activation of 229E S protein [130]. In addition, cathepsin L was reported to cleave spike (S)-protein of the Severe acute respiratory syndrome (SARS) coronavirus [131]. The cleavage and the activation of the viral S protein by cathepsin L induced a conformational change in the S-protein required for the binding to the cellular receptor, angiotensin-converting enzyme 2 (ACE2) [131]. The activation of S protein involves at least two consecutive cleavages by host cell proteases and is essential for viral infectivity [132]. Although ACE2 is a cellular receptor for two divergent coronaviruses, SARS coronavirus and human coronavirus NL63, it was reported that the inhibitors of cathepsin L blocked the infection only by SARS, but not by NL63 virus [132]. In addition, expression of exogenous cathepsin L significantly enhanced infection mediated by the SARS S protein, but not by the NL63 S protein or the vesicular stomatitis virus G protein [133]. SARS infections emerged in 2003 affecting >8000 persons and resulting in death in ∼10% of cases [134]. Coronaviruses still represent a leading source of novel viruses for emergence into the human population [132]. Recently, several neutralizing antibodies have been developed and some of them showed promising results in the therapy of non-human primates [135]. However, the combinational therapy with antibodies and inhibitors could have several advantages.

Paramyxoviruses are enveloped, single-stranded, negative-sense RNA viruses which include a number of major human pathogens, such as measles virus, mumps virus, human respiratory syncytial virus, and Hendra and Nipah virus [136]. Proteolytic activation of the fusion protein of the Nipah virus is a prerequisite for the production of infectious particles and for virus spread via cell-to-cell fusion [136]. Recently cathepsins B and L were reported to be required for the cleavage and productive replication of pathogenic Nipah virus, but not Hendra virus [137].

Reoviruses form non-enveloped, double-stranded RNA viruses. The virus entry into cells is initiated by the attachment of virions to cell surface receptors [138] and by receptor-mediated endocytosis [139]. In the host cell endolysosomes, virions undergo stepwise disassembly, forming discrete intermediates, the first of which is the infectious subvirion particle [139]. A recent study examined a contribution of individual cathepsins B, L and S to the virus spread in newborn mice [140]. In was shown that the survival rate of cathepsin B-deficient mice was enhanced in comparison to that of wild type mice, whereas the survival rates of cathepsin L and cathepsin S deficient mice were weakened. Virus titers at sites of secondary replication in all strains of cathepsin-deficient mice were lower than those in wild type mice, indicating that all cathepsins could participate in the spread of the virus. Clearance of the virus was delayed in cathepsin L- and cathepsin S-deficient mice in comparison to the levels for wild type and cathepsin B- deficient mice, as a consequence of the important functions of the two cathepsins in immune response [140]. The study shows that the functions of proteinases in the virus entry into the cell as well as in host immune response are relevant for the possible therapy with inhibitors.

## CONCLUSIONS AND FUTURE DIRECTIONS 

During the last decade, our understanding of both adaptive and innate immune responses has greatly increased. Cysteine cathepsins were shown to play some unexpected, yet not completely understood roles in the endosomal TLR activation and in the NLRP3 inflammasome activation. The role of endogenous inhibitors as well as pathogen proteinases and inhibitors in this process is still elusive. Although protease inhibitors have a potential use as therapeutics in virus infections, the effects on innate and adaptive immune response should not be underestimated. The understanding of the mechanisms by which proteinases and inhibitors used by the pathogens interfere with the host adaptive and innate immune response is essential for the development of therapeutic inhibitors. 
